# Adjustment of Clasps

**Published:** 1850-10

**Authors:** 


### ARTICLE VII.

### *Adjustment of Clasps.*

It has been justly remarked, in a late number of the "Journal," that clasps cannot be entirely dispensed with, in the insertion of partial sets of artificial teeth. Properly constructed atmospheric plates, have, to some extent, superseded the necessity of using them; but still, in a great many cases, they are undoubtedly quite indispensable. The objections to their use are very much lessened by their perfect adjustment to the teeth to which they are applied. To effect this object, it is, of course, necessary that we should have something more to depend upon, than the ordinary plaster model from which the plate is made. A number of plans, more or less useful, have been suggested for this purpose. That which I have found most satisfactory is, Fogle's strip, for confining temporarily the clasp to the plate; but I have lately fallen upon an exceedingly simple method, which, so far as I have tried it, has most perfectly answered the desired purpose. I do not know whether it is new, (it is certainly so to myself and to those to whom I have mentioned it,) or whether its success in the few cases in which it has been tried, has led me to over-estimate its advantages.

In taking the impression, I am only careful to secure a perfect impression of that part of the mouth to which the plate is to be fitted. After the plate is made and found to fit, it is fixed in its proper place in the mouth, with a little Venice turpentine, placed on the upper surface, the mouth being previously wiped dry. An impression is now taken and the plate withdrawn from the mouth with the wax. The Venice turpentine, as will be seen, serves to keep the plate in place during the process of taking the impression, but does not prevent it from leaving the mouth when the wax is withdrawn. From this impression a plaster model is made, which will show the exact relation of the teeth to which the clasps are to be fitted and the plate. With a model made in this way, I do not hesitate at once to solder on the clasps permanently. I am always in the habit of forming the clasps upon castings made from impressions carefully taken of the teeth to which they are to be attached.

It may be asked: What advantage do you gain by taking a second impression with the plate in the mouth? Why will not the first impression, if correctly taken, answer quite as well? For the same reason, that the teeth of that impression cannot be depended upon for the perfect formation of the clasps. It is so important that the portion of the mouth, which is to receive the plate should be accurately taken, that is very difficult, if not quite impossible, to get at the same time a perfectly correct impression of the adjacent teeth. But after the plate is made, the attention and efforts can be exclusively directed toward obtaining a correct impression of the teeth to which the clasps are to be attached, which must be comparatively easy. It is, however, quite unnecessary to discuss this question; for the method suggested is so easily tested, that the matter may be at once settled, as such thing only can be, by active experiment, if the advantage to be gained is thought worthy of the trial.

A.

Washington City, D. C., *Oct.*, 1850.

